# Molar and Incisor Hypomineralization

**DOI:** 10.31729/jnma.6343

**Published:** 2021-03-31

**Authors:** Basim Almulhim

**Affiliations:** 1 Department of Preventive Dental Sciences, College of Dentistry, Majmaah University, Al-Majmaah, Saudi Arabia

**Keywords:** *developmental defects*, *enamel hypoplasia*, *hypersensitivity*, *molar incisor hypomineralization*

## Abstract

Molar and incisor hypomineralization is a developmental defect that is systemic in origin that affects one or more than one permanent first molars, and is often associated with permanent incisors. It is usually characterized by well demarcated opacities and qualitative enamel defects caused by decreased inorganic enamel components, and reduced mineralization. It can cause esthetic, functional, psychological, and behavioral problems in children. Its reported prevalence varies widely, from 2.5% to 40.2%. Multiple aspects of dental treatment for it are challenging, such as behavior management, difficulty in achieving adequate local anesthesia, tooth hypersensitivity, and retention of restorations. This review discusses the most important considerations pertaining to its prevalence, severity, etiology, differential diagnosis, and some of the challenges and treatment modalities applicable in young patients. Data is collected from PubMed, Medline, and Embase databases.

## INTRODUCTION

Molar and incisor hypomineralization (MIH) is a systemic developmental defect affecting one or more permanent first molars (PFMs) and is frequently associated with permanent incisors. It is characterized by well demarcated opacities; qualitative defects of enamel caused by reduced inorganic enamel components; and reduced mineralization that can cause esthetic, functional, psychological, and behavioral problems in children.^1-3^ Usually, no other permanent teeth are involved.

Studies of the prevalence of MIH have shown variable results, with reported rates ranging from 2.5% to 40.2%.^4-6^ According to one study, the condition affected one in six children in the general population.^7^ As the PFMs are the first permanent tooth to erupt, they may exhibit rapid caries progression and be more prone to break down than teeth that erupt later.^1-8^ The dental management of MIH presents substantial challenges in numerous respects.^9-11^

This review discusses the most important aspects of MIH, including factors pertaining to its prevalence, etiology, severity, differential diagnosis, and treatment modalities in young patients.

## ETIOLOGY

To date, the causative mechanism of MIH remains unknown, but some authors consider it as multifactorial.^1,10,12-14^ Congenital and environmental factors have been proposed,^1,10,12,14-17^ as has the involvement of systemic conditions, such as respiratory tract infections. Other suggested causes include childhood diseases,^1,2,12^ particularly those requiring the extended use of antibiotics,^1,16^ as well as other medical conditions that may influence or disrupt amelogenesis during the early development of the PFMs.^16^ In one study, the environmental pollutants polychlorinated dibenzo-p-dioxins/dibenzofurans were associated with MIH,^18^ although notably, in a more recent study, no such significant association was found.^19^ Exposure to contaminants as polychlorinated biphenyls is associated with an increased risk of MIH.^17,20^ The causes of molar and incisor hypomineralization (Table 1).

**Table 1:** Causes of Molar and Incisor hypomineralization.

Congenital	Hereditary factors that are involved in the etiology of MIH may interact with systemic factors^1,20,21^
Environmental Factors	Exposure to pollutants (dioxins) during third trimester and/or first three years of life^1,10,12,14-17,19-21^
Systemic Conditions	Disruption of amelogenesis during early maturation^2^ caused by: Respiratory tract infections^1,2,13^ Perinatal complications^1,2,13,15,16^ Oxygen deprivation^1,2,13,15,16^ Low birth weight^1,2,13,15,16^ Calcium and phosphate metabolism disorders^1,2,13,15,16^ Recurrent childhood illnesses^1,2,13,15,16^ Prolonged use of antibiotics^1,17^ Long-term breast feeding^1,2,13,15,16^

## SEVERITY

A severity scale for individual teeth is defined that categorizes MIH as mild, moderate, or severe (Table 2).^21^

**Table 2:** Molar and incisor hypomineralization severity scale.^21^

	Mild	Moderate	Severe
Crown appearance	Demarcated opacities^*^ not involving the load-bearing area of the molars	Intact atypical restorationt^†^	Post-eruptive enamel breakdown^‡^
Enamel loss	Isolated opacities	Involvement of occlusal or incisal 1/3 of teeth, but without initial post-eruptive enamel breakdown	Post-eruptive enamel breakdown, usually severe
Caries	No associated caries	Caries limited to one or two surfaces and without cuspal involvement, and possible post-eruptive enamel breakdown	Substantial progression of caries
Sensitivity	Normal dental sensitivity	Child usually exhibits normal dental sensitivity	A history of dental sensitivity
Esthetics	No parental concerns	Parental concerns	Parental concerns

* A demarcated defect presenting as a change in enamel translucency; variable in degree. Enamel is usually of normal thickness and has a smooth surface. It can be yellow, brown, or white in color.

† Restorations on the posterior teeth that extends to the buccal, palatal, or lingual side. Some opacities at the borders of the restorations are common. Facial restorations not related to dental trauma are evident on the anterior teeth.

‡ A defect which indicates loss of tooth surface post eruption. Pre-existing demarcated opacities are often associated with enamel loss.

The Würzburg MIH workgroup recently proposed the MIH Treatment Need Index (MIHTNI), which can be used for diagnosing and treating MIH in individual teeth.^22^ The MIHTNI score is usually not dependent on the degree of destruction, but if the tooth is hypersensitive, it is considered.^22^ The index values of molar and incisor hypo-mineralization (Table 3).

**Table 3:** Molar and incisor hypomineralization treatment need index.^22^

Score	Definition
**0**	No MIH
**1**	MIH without hypersensitivity and without defect
**2**	MIH without hypersensitivity, but with defect
**2a**	< 1/3 defect extension
**2b**	> 1/3, but < 2/3 defect extension
**2c**	> 2/3 defect extension and/or defect close to the pulp requiring extraction or atypical restoration
**3**	MIH with hypersensitivity, but without defect
**4**	MIH with hypersensitivity and with defect
**4a**	< 1/3 defect extension
**4b**	> 1/3, but < 2/3 defect extension
**4c**	> 2/3 defect extension and/or defect close to the pulp requiring extraction or atypical restoration

## DIAGNOSIS

Diagnosis of MIH can be challenging, and the condition may be confused with other hereditary conditions, particularly developmental enamel defects as amelogenesis imperfecta, fluorosis, white spot lesions, enamel hypoplasia, and traumatic hypomineralization. The criteria for diagnosis of MIH are based on the clinical findings of well demarcated opacities, breakdown that is post eruptive, atypical restorations, and PFM extraction for reasons suggestive of MIH.^1,3,21,23-30^ The factors that should be considered in the differential diagnosis of MIH (Table 4).

**Table 4:** Differential diagnosis of MIH.

**Molar and incisor hypomineralization^1,3,21,29,30^**	White, creamy, or yellow-brown opacities Affects one or more than one first permanent molars and often associated with permanent incisors, while other teeth are not affected Lesion > 1 mm Asymmetrical pattern Caries often present Post-eruptive enamel breakdown
**Amelogenesis imperfecta^1,23-26^**	Often with a family history Affects primary and permanent dentitions Possibility of enamel resorption and ankylosis Possibility of anterior open bite Possibility for agenesis of second molars
**Fluorosis^1,3,21,27,28^**	With a history of fluoride intake during tooth development Primary dentition is usually not affected, but all permanent teeth usually tend to be involved Symmetrical and bilateral pattern Caries resistant
**White spot lesions^1,3,21,30^**	Occur in the cervical areas of teeth because of plaque accumulation in this area
**Traumatic hypomineralization^1,3,21,30^**	History of injury to the affected deciduous tooth Often limited to one tooth Asymmetrical pattern

## CHALLENGES AND CLINICAL PROBLEMS ASSOCIATED WITH MIH

Tooth sensitivity can cause a child to neglect oral hygiene, resulting in susceptibility to caries. There is a need for early emphasis on preventive measures to avoid post-eruptive enamel breakdown. The condition can be associated with chronic pulpal inflammation, and hence, adequate local anesthesia can be difficult to achieve. Inhalation sedation with additional supplemental anesthetic may decrease pain during dental treatment. The retention of dental restorations is a substantial problem and may necessitate pretreatment of the enamel with 5% sodium hypochlorite that removes the proteins encasing the hydroxyapatite crystals. Esthetic concerns are common, especially in cases involving the anterior teeth. Tooth loss can result from aggressive progression of caries. Some cases require the use of special management techniques for addressing dental fear and/or anxiety resulting from stories heard from parents or peers or from pain experienced during a previous dental visit. Inhalation sedation can be used to manage dental fear and anxiety during dental treatment. Other challenges that may arise include those related to quality of life, such as difficulties in treating children with no known previous dental visits, need for long and/or multiple appointments, need for missing school for some days, diminished oral health-related quality of life along with higher treatment costs as treatment done under general anesthesia.^11,31-32^

## TREATMENT MODALITIES

Early identification and diagnosis of patients that are at risk for MIH can facilitate the provision of more effective treatments, better results, and reduced treatment costs.^33,34^ Many factors must be considered before deciding a specific treatment protocol, including the child's age, severity of the MIH, restorability of tooth or teeth involved, presence or absence of pulpal involvement, presence of third molar germs, long-term prognosis, and cost of treatment.^32^ Administration of Local anesthesia is often challenging in children with MIH, and it can be rendered more difficult to achieve because of chronic pulpal inflammation. The tooth could be highly sensitive to hot and cold temperatures.^35^ As teeth affected by MIH could be more difficult to anesthetize, thereby the use of inhalation sedation (N_2_O) to increase the pain during dental treatment^33,36,37^ and use of supplemental anesthetic techniques, such as intraosseous, palatal, and/or intraligamental anesthesia have been suggested.^35,38^

Articaine infiltration can be effective, and inferior alveolar nerve block adjunct with buccal articaine infiltration has been shown to be more effective.^39^ Rubber dam isolation and the application of saliva ejectors to replace high-volume suction can also be helpful in the management of teeth hypersensitivity.^1^ William, et al.^33^ have described useful approaches for the management of children with MIH.

1. **Risk identification**
  Assess caries risk and the patient's medical and dental history.
2. **Early diagnosis**
  Examine the patient as early as possible, identify and assess molars at high risk of MIH via dental radiographs before eruption, and monitor those teeth during eruption.
3. **Remineralization and desensitization**
  Emphasize the benefits associated with practicing adequate oral dental hygiene measures at home, including the use of fluoridated toothpaste and with the professional application of remineralizing agents as topical fluoride varnishes. Casein phosphopeptide-amorphous calcium phosphate can be helpful in reducing dental sensitivity, particularly in hypocalcified areas.^33,40^
4. **Prevent dental caries and post-eruptive breakdown**
  Emphasize the importance of adequate oral hygiene strategies, desensitizing agents, reducing cariogenic habits, and professional application of pit and fissure sealants in the dental clinic. Remind child's parents that soft drinks generally contain considerable amounts of sugar^8^ and hence, should be avoided.
5. **Restorations or extractions**
  Esthetic concerns involving anterior teeth with MIH can be managed via different techniques, such as microabrasion and bleaching. Bleaching may be advisable in full-thickness brownish-yellow or yellow defects, but not in whitish-cream or creamy-yellow defects that are located in inner part of the enamel. Resin infiltration may be more effective, especially in areas with shallow defects.^33,40-43^ However, defects involving the entire width of enamel may require conventional approaches, such as composite resin restorations, particularly if the carious process has involved one or more surfaces with using self-etching adhesive. Full coverage restorations, such as stainless-steel crowns are good options for teeth with severely damaged surfaces. The rates of failure and replacement of restorations are very high; thus, long-term follow-up is mandatory.^33,40,44^ Some authors have suggested the pretreatment of enamel with 5% sodium hypochlorite that removes protein encasing the hydroxyapatite crystals.^45,46^ The removal of remaining hypomineralized enamel before the placement of composite restorations has been recommended.^44,47,48^ Some restorative materials, such as glass ionomer cement (GIC) or resin-modified glass ionomer cement (RMGIC) are not recommended to be used in occlusal surfaces of hypomineralized molars (load-bearing areas), but can be used temporarily to reduce hypersensitivity (for 1 to 2 weeks), until a definitive restoration can be placed. They can be used in partially erupted teeth.^49^ GIC restorations have various advantages, including easy placement, fluoride release, and chemical bonding properties. RMGIC restorations have similar advantages to GIC restorations, but are superior in terms of ease of handling, wear resistance, fracture toughness, and resistance.^50-52^
  If restoration of the tooth is impossible, and extraction is the only treatment option, early orthodontic assessment is recommended prior to extraction, with close monitoring of the development of the occlusion.^53-56^ Extraction may be the treatment of choice for non-restorable teeth or those with poor prognoses, but in very young children, occlusal guidance must be utilized to ensure that the second permanent molars move in position of the first molar.^33,40,53-57^ Many factors must be considered, especially in cases involving the mandibular arch. The optimal timing for extractions is evidently between the ages of 8 and 10 years.^55^
  Extractions should be performed after the eruption of the lateral incisor (age 7 years), but usually before eruption of the second permanent molar and the second premolar. Radiographs should be used to confirm that the second permanent molar is within the bone, and that calcification of the bifurcation area has commenced 55. If the extractions are performed too early, the second premolars can drift distally, particularly in the mandible, and the labial segments can retrocline, thus increasing the overjet. In addition, early extractions do not permit until to confirm the presence of third molar follicles prior to the commencement of the extractions. If the extractions are performed are delayed, the second premolar teeth may tip distally, and spaces between teeth might arise. In addition, occlusal forces can cause mesial tipping and rotation of the second molars.
  The Angle's classification of occlusion must be determined prior to PFM extraction, and compensation or balancing may be required. In patients with Class I occlusion and minimal crowding, the maxillary and mandibular PFMs should be extracted at the optimal time, and compensatory extractions should be performed when extractions are restricted to the mandibular PFMs.^55,56^ In patients with Class I occlusion and moderate crowding, simultaneous extraction of both maxillary and mandibular PFMs should be performed at the optimal time, but crowding of the permanent teeth would require adequate management later. If bilateral buccal segment crowding is present, the balancing and compensatory extractions of maxillary PFMs may be required. In cases of labial crowding, delaying the PFM extraction until the eruption of permanent second molars should be considered, to facilitate the utilization of the extraction space for alignment with a fixed appliance.^55,56^ Class II malocclusion with minimal crowding is difficult to manage, and orthodontic consultation should be sought, ideally. The maxillary and mandibular PFMs should be extracted at an optimal time; however, crowding of the permanent teeth will require adequate management later. PFM extraction should be delayed, if possible, to facilitate utilization of the extraction space for correcting the incisor relationship via a fixed appliance, especially in the maxillary arch.^55,56^ Class II malocclusion with moderate crowding is also difficult to manage, and orthodontic consultation should be sought. The maxillary and mandibular PFMs must be extracted at an optimal time, and the space obtained should be utilized immediately for the relief of crowding via treatment with fixed orthodontic appliances.^55,56^ Dental management of Class III malocclusion is challenging, and orthodontic consultation should be sought. Neither balancing nor compensatory extractions are generally recommended.^55^ Treatment options are summarized (Table 5).
6. **Maintenance**
  Long-term follow-up with close monitoring of the restoration margins is required to avoid posteruption breakdown. In the long term, full coronal coverage restorations should be considered. Patients who have undergone extractions must be closely monitored by an orthodontist.
  MIH is frequently associated with permanent incisors and affects one or more first molars and is characterized by well demarcated opacities and qualitative defects of enamel caused by reduced inorganic enamel components, and reduced mineralization that can cause esthetic, functional, psychological, and behavioral problems in children. The prevalence of MIH is increasing. The causative mechanisms involved remain unclear, but they are thought to be multifactorial. Children who have systemic problems during the first three years of life or are born preterm and/ or children whose mothers have positive medical history during the pregnancy may develop MIH. The dental management of children with MIH is more difficult and challenging for general dentists and pediatric dentists. The early diagnosis of children with MIH and monitoring their PFMs would provide better treatment results. More emphasis on oral hygiene strategies, prevention, and remineralization processes are required to reduce dental hypersensitivity.

**Table 5:** Treatment options for molar and incisor hypomineralization.^21,33,40-43,47,49,54-56^

**Anterior teeth**	Treatment decisions are based on the severity of the condition: Resin perfusion Microabrasion Bleaching Resin Infiltration Resin composite restorations Composite veneers
**Posterior teeth**	Treatment decisions are based on the severity of the condition: Desensitizing toothpaste Fluoride varnish Sealants GIC or RMGIC as temporary restorations (1-2 weeks) to reduce tooth sensitivity Resin composite restorations Full-coverage restorations (for example, stainless steel crowns) Extraction
